# Seeking and sharing: why the pulmonary fibrosis community engages the web 2.0 environment

**DOI:** 10.1186/s12890-016-0167-7

**Published:** 2016-01-12

**Authors:** Karen Albright, Tarik Walker, Susan Baird, Linda Eres, Tara Farnsworth, Kaitlin Fier, Dolly Kervitsky, Marjorie Korn, David J. Lederer, Mark McCormick, John F. Steiner, Thomas Vierzba, Frederick S. Wamboldt, Jeffrey J. Swigris

**Affiliations:** Department of Community and Behavioral Health, Colorado School of Public Health and the Adult & Child Center for Health Outcomes Research and Delivery Science (ACCORDS), University of Colorado School of Medicine, CO University of Colorado Anschutz Medical Campus, Aurora, CO USA; Department of Sociology and Criminology, University of Denver, Denver, CO USA; Department of Pediatric Infectious Disease, University of Colorado School of Medicine, Aurora, CO USA; Participation Program for Pulmonary Fibrosis (P3F), National Jewish Health, Denver, CO USA; PF Strategies, LLC, Black Hawk, CO USA; Columbia University Medical Center, New York, NY USA; Kaiser Permanente, Aurora, CO USA; Interstitial Lung Disease Program, National Jewish Health, 1400 Jackson Street, Denver, CO 80206 USA

**Keywords:** Pulmonary fibrosis, Internet, Web forum, Online health information, Blog, Caregiver

## Abstract

**Background:**

Pulmonary fibrosis (PF) is a rare, progressive disease that affects patients and their loved ones on many levels. We sought to better understand the needs and interests of PF patients and their loved ones (collectively “reader-participants”) by systematically analyzing their engagement with the World Wide Web (the current version referred to as Web 2.0).

**Methods:**

Data were collected from three PF-focused, interactive websites hosted by physician-investigators with expertise in PF. All data generated by reader-participants for approximately 10 months were downloaded and then analyzed using qualitative content analysis methods.

**Results:**

PF experts posted 38 blog entries and reader-participants posted 40 forum entries. Blogs received 363 responses, and forum entries received 108 responses from reader-participants. Reader-participants primarily used the three websites to seek information from or offer a contribution to the PF community. Information was sought about PF symptoms, diagnosis, prognosis, treatments, research, pathophysiology, and disease origin; reader-participants also made requests for new posts and pleas for research and sought clarification on existing content. Contributions included personal narratives about experiences with PF, descriptions of activities or behaviors found to be helpful with PF symptoms, resources or information about PF, and supportive comments to other PF sufferers.

**Conclusions:**

PF patients and their loved ones engage the Web 2.0 environment at these PF-focused sites to satisfy their needs to better understand PF and its impacts and to support others facing similar challenges. Clinicians may find it beneficial to encourage PF patients’ involvement in internet forums that foster dynamic, bi-directional information sharing.

**Electronic supplementary material:**

The online version of this article (doi:10.1186/s12890-016-0167-7) contains supplementary material, which is available to authorized users.

## Background

Pulmonary fibrosis (PF) is a chronic, typically progressive and often life-shortening condition. Patients with PF suffer from intrusive symptoms and impaired quality of life [1], and their loved ones struggle to adapt to their roles as caregivers [2]. Over the last decade, investigators have made great strides in deciphering PF pathogenesis, and for the first time ever, pharmacological therapies are approved to treat certain forms of PF. However, there is no cure, and patients continue to die from and endure impairments induced by PF.

Like people with other medical conditions, many patients with PF (and their loved ones) are hungry for information about the disease [3] and desperate for support from other people facing similar challenges. The current generation of the World Wide Web (Web 2.0) fosters dynamic, bi-directional, open information sharing and collaboration among communities of users through blogs, forums and many other web services (www.Webopedia.com). The explosion of online health-related resources in today’s Web 2.0 environment gives patients and their loved ones limitless opportunities to access information, share personal experiences, and give and receive support [4].

For researchers, the internet provides a vast repository of rich accounts of illness experiences [5] that can be examined to enhance understanding of how patients and their loved ones are affected by disease and to shed light on their needs, values and judgments. Synopsizing such information can provide practitioners who care for these patients with vital information that could be used to foster improved communication with patients and their families and ultimately pave the way to a more fully patient-centered approach to care.

The objective of this study was to analyze posts from three websites developed independently by two PF expert clinician-investigators. The websites were created to raise awareness, educate, and provide reader-participants with platforms for exchanging information and experiences. We sought to better understand the needs and interests of PF patients and their loved ones by systematically analyzing their engagement with the Web 2.0 environment at these sites.

## Methods

### Data collection

Data were collected from the websites for approximately 10 months. Two websites are organized as blogs: narratives on topics related to PF are posted, and readers have the option to respond (or not). Responses are not constrained to the blog topic. The third website is organized as a forum in which readers may contribute original posts and/or respond to previously posted material. The forum and one of the blogs are facilitated by J.J.S. The second blog is facilitated by D.L. All three websites were launched in August 2013 and continue to operate. For this study, online data on the poster or responder (e.g., user name, date of post) and the content of all posts and responses from launch through early May 2014 were downloaded from each website and managed within Microsoft Excel spreadsheets. Because all data collected and analyzed are online and freely available to the public, this study was exempted from ethics board approval (National Jewish Health Institutional Review Board; HS#2960), and written, informed consent was neither obtained nor required. Please see Additional file 1: Online Supplement for more details.

### Data analysis

Data were analyzed in an iterative process using qualitative content methods and reflexive team analysis [6, 7]. The contents of each spreadsheet were read multiple times by K.A., T.W., and J.J.S. to achieve immersion. Code categories were developed for all data (posts and/or responses) generated by reader-participants using an emergent, rather than a priori, approach to emphasize respondent perspectives and de-emphasize team speculations [7]. Content generated by the facilitators was not coded.

Data were independently coded by K.A., T.W., and J.J.S. and then discussed until consensus was achieved [7]. Consensus codes were then applied to the data. Nine main content code categories emerged; multiple codes were frequently assigned to entries due to the complexity of their content [7]. The study team met regularly to check new findings, to discuss emergent new codes, subcodes, and themes, and to assess the preliminary results of the analysis process [8].

## Results

During the data collection period, 38 blog entries and 40 forum strings were posted. Blog entries received a collective total of 363 responses from 149 unique user names. Forum strings were started by posts from 15 unique user names and 8 unnamed reader-participants, and received a collective total of 108 responses from 26 unique user names. These responses represent only a fraction of page views. Traffic data analytics are available only for the blog and forum facilitated by J.J.S. During the study period, the main blog page received 9600 views, and individual entries received between 130 and 3752 views; the main forum page received 5964 views, and the individual strings received between 125 and 2037 views.

Content generated by reader-participants included personal narratives about their own PF experiences or those of their loved ones; questions seeking information about PF-related topics; expressions of gratitude for the blog(s), a specific post, or a reply; descriptions of activities or behaviors personally found to be helpful with PF symptoms; sharing of resources or information about PF; supportive comments from one PF sufferer to another; requests for new or additional PF-related posts or research; clarification questions about post content; and “other”, a category largely comprised of casual comments not relevant to PF, or polite remarks concluding a thread. The frequencies of these content code categories are presented in Table 1. Of the total, 42 comments came from patients’ loved ones. The distribution of code categories for this subgroup mirrored Table 1.

**Table 1:** Content generated by reader-participants

Content type	Number
Personal narratives about own PF experiences or those of loved ones	264
Questions seeking information about PF-related topics	181
Expressions of gratitude for the blog(s), a specific post, or a reply	179
Descriptions of activities or behaviors personally found to be helpful with PF symptoms	64
Sharing of resources or information about PF	62
Supportive comments from one PF patient to another	41
Requests for new or additional PF-related posts or research	28
Clarification questions about post content	11
Other (casual comments not relevant to PF and polite remarks concluding a thread)	78

### Content analysis

Content analysis indicated that reader-participants primarily used the websites for one or more of the following three purposes: 1) to share their personal narratives about PF; 2) to seek information from the PF community; and 3) to offer contributions to the PF community. Each purpose is described in further detail below. The substantively irrelevant content categories “expressions of gratitude” and “other” have been dropped from further discussion. All quotations that appear below may be shortened for clarity (where indicated) but are otherwise presented with fidelity to their original form, including emphases, misspellings, and grammatical errors.

#### Sharing personal experiences

Reader-participants most commonly used the websites to share personal narratives about their own and/or their loved ones’ experiences with PF. The primary intention appeared to be to provide psychosocial support. These narratives often embedded inspiration within personal experience, as in the following blog response from a spouse of a PF patient:

*My husband of 44 years has had PF for 9 years. From the start we have been in this together as in everything else in our life…Our house is called “Hope Cottage”. We do all this together with the help of all our health professionals including hospice support who have always been absolutely upfront and honest. Wraparound care is so important. The websites also help us both in sharing and support. This disease affects everyone close to the sufferer, therefor everyone should be involved.*

However, some narratives about personal experiences seemed primarily intended to provide context for a question to which they hoped for answers:

*i have been diagnosed about 1 year plus i had a real bad cough which let up but now it’s terrible nothing seems to work. i was told i have to live with it…please tell me there is a treatment.*

#### Seeking information

The second most common purpose of posts and responses was to ask questions, as reader-participants primarily sought information about a wide range of PF-related topics, including symptoms, diagnosis, prognosis, treatments, research, pathophysiology, and disease origin. The specific types of information sought are presented in Table 2. Sometimes this was accomplished with a relatively straightforward question, such as “How close are researchers to finding a gene marker, to identify the hereditary factor related to this disease?” or “What is the connection between prednisone and having a biopsy?” Other entries included much more description and, often, multiple questions:

**Table 2:** Information sought by reader-participants

Type of information sought	Number	Example post
Pathophysiology	43	What usually causes a exacerbation in IPF? Mine always follow, by several weeks, being sick with a cold that does or not does go to my lungs. We have been treating the exacerbation as inflammation and since I respond so well to using prednisone at that time, that would seem to confirm the inflammation… if IPF isn’t inflammation, why would a cold now trigger inflammation and is inflammation a normal response in the lungs to a cold?
Signs/symptoms	39	I was diagnosed with lung cancer in August of 2012 using a CT scan (15 months ago). In March 2013 I was diagnosed as having IPF from a follow up CT scan for the lung cancer issue (8 months ago). I have no symptoms of lung cancer or IPF to date. I have a question for you if you can answer for me. How long after being diagnosed for there diseases, should I expect to develope symptoms?
Medicinal/pharmaceutical treatment	37	Is Cyclophosphamide more likely than Imuran or Cellcept to produce drug induced megloblastic or pernicious anemia. I have LcSSc and have been on a 50 mg daily dose of Azathioprine now since 2005. I was on prednisone for only 6 months in 2005, but none since then. Last year I developed pernicious anemia and am suspicious that Azathioprine may have contributed to the condition. I also had a laproscopic partial fundoplication procedure, and am aware that development of pernicious anemia after the procedure is a possibility. My hematologist indicates I have an inadequate amount of intrinsic factor, and regular B12 injections have returned my CBC counts to normal levels. Would Cyclophosphamide make the pernicious anemia problem worse?
Diagnoses and diagnostic process	36	I have a diagnosis of LcSSc (Limited Cutaneous Systemic Sclerosis) with a UIP pattern of Interstitial Lung Disease. My Rheumatologist and Pulmonologist use different terminology when defining my diagnosis. The American Thoracic Society and the American College of Rheumatology may have different diagnostic criteria, which may explain the difference. I have heard my diagnosis described as LcSSc with secondary ILD-UIP pattern. Based upon exact wording used in the diagnostic criteria, what is the diagnosis for a patient with LcSSc who also has Pulmonary Fibrosis with a UIP pattern.
Supplemental oxygen	24	I’ve asked my Pulmo about using O2 supplementation so that I can maintain a better excercise program, but he continues to maintain that I am not in need of O2 supplementation at this time. I’ve been trying to figure out why my body makes me quit exerting before my O2 level desaturates below 90 %. I asked my Pulmo about having an exercise stress test done but he doesn’t seem to interested in the idea. I have also been diagnosed with Scleroderma, and am wondering if the blood vessel constriction due to the collegen vascular problems can account for why my O2 levels don’t desaturate, even though my muscles feel like they are deprived of O2? Could supplemental O2 help with this situation and allow me to exercise more?
Behavioral/non-medicinal treatment	15	I was diagnosed with IPF in May 2013 - since modern medicine really has nothing to offer me in terms of treatment, I have been working with a natural therapist, as well as a 105-year old retired osteopath and physicist, who has been researching natural (Native American) therapies for over 60 years. My treatment includes natural anti-inflammatories, natural immune system support supplements, hydro-colon therapy, and an extremely high alkaline diet, with no wheat, no dairy, no sugar, no caffeine, and no alcohol. My follow up with my pulmonologist showed a "marked improvement" in lung function. What are your thoughts regarding diet and natural therapies?
Research	13	I seem to remember that you shared some information with me at one of our support group meetings in the summer…about a Michigan study on desaturation during the 6MWT (six minute walk test) which showed that those patients who desaturated during the test had a higher mortality rate than the patients that did not desaturate. The study concluded that the test seems to be the best predictor of longevity; better than Spirometry or PFT. Did I get that right…was there more news on that?
Lung transplantation	11	I do have a question about the transplant process. In 2009 I had a lung biopsy and told I had IPF I was also told I had 2 years to live unless I had a double lung transplant. Also told to make my funeral arrangements in case I didn't live long enough to get my transplant. I went through the process and told I needed to loose 6pds. to be listed. The visit I had to return when loosing the 6pds. I was told I didn't need a transplant at this time I was too healthy. I was 54 y.o. at that time and had high hopes of returning to my productive life. Now I am in limbo not healthy enough to do daily routines yet too healthy for a double lung transplant. Do you have any input on this kind of decision making? Thank you for taking time to respond to this question. I am sure I am not the only person out there that this has happened too.
Progression/prognosis of disease	9	is there any connection between age (my age) an how many years left to live with ipf ?
Administrative/community issues	7	I believe it's Courtney that keeps the PFF Support Group list up to date. However, your list is only for the US. Does PFF have any plans to incorporate a list for other countries such as Canada?
Origin of/explanation for disease	5	I was diagnosed with ILD this summer through a bronchoscopy and hi-res cat scan, and consider my case mild to moderate, for which I am grateful. I wondered as I read your blog — has there been any research into the relationship of dust mites and their waste to PF? For years I slept on the same mattress unaware of this problem. It would seem to me that their environmental “leavings” could be as much a problem as other animal dander and poo.
Treatment centers/referrals	4	As it is virtually impossible to obtain the stem cell treatment for IPF in the USA at this time, is there anywhere out of country where you would feel comfortable in having this treatment done?
Other	5	Any idea of how many home O2 concentrators are sold each year?

*Question – I am fortunate in that I have always been very active. Over the years I’ve worked with a physical trainer, taken many different aerobic classes, biked long distances, etc. Currently, after being diagnosed with IPF, I am working with a trainer, walking a lot and spinning. As a result, my exercising is more vigorous than what might take place in pulmonary rehab and I’m assuming that’s why my pulmonologist has not prescribed it for me. My question is, isn’t there more than just exercise that would be beneficial for me in pulmonary rehab? Support from other PF patients? Saturation monitoring that is better than I can do with my little oximeter (Have you ever tried to keep one of those things on your finger while you’re throwing around a medicine ball?) Information from staff? Why wouldn’t it be good for someone like me be able to attend pulmonary rehab? Thanks!*

The websites were used to ask clarifying questions about content in facilitator-generated posts or replies that were unclear. For example, one reader-participant responded to another’s post with the question “What is a Center of Excellence?,” while another asked a facilitator “What’s the acronym NSIP stand for?” Many reader-participants also made requests for or indicated interest in new or additional PF-related posts. As one put it:

*I look forward to future posts discussing some of the very real risks [of lung transplantation] and how we as patients can work with our medical team to minimize those risks such as obesity.*

#### Offering a contribution

Reader-participants sought to contribute a considerable range of resources to others in the PF community. Perhaps most notably, they shared descriptions of activities or behaviors they had found to be personally helpful with PF symptoms. These descriptions appeared intended to introduce ideas to other readers about approaches they might consider, and often included advice. This is evident in the following reply to a blog post about intimacy with PF:

*[Sex] was a bit of a concern with my husband was on full time oxygen. First of all, his desire for sex decreased. I am a bit younger but still in my 50′s – not 30′s. I understood and didn’t care for the most part but did miss the intimacy. When we did – we found that the non lung care patient should do most of the work – so to speak. I don’t know how to say it without being too blunt – but be on top and be careful of putting too much pressure on the chest of the one with the lung issues. He did not wear oxygen during our “intimacy” but I would not have minded if he had. Fortunately, he was never compromised. Hope this helps someone.*

In addition, reader-participants frequently shared resources or information about PF that they had found elsewhere. For example, one forum reader-participant posted the following:

*I attended the inaugural webinar [on pulmonary wellness] tonight and was extremely impressed by the level of information provided. Even though much of it was already covered in my pulmonary rehab, it provided an excellent overview… on what pulmonary rehab should be and answered many questions that frequently come up on this forum. This is another doctor who is blogging and providing webinars to meet the growing need of patients with lung disease. He will be doing a monthly webinar and the next one is Breathe Deep! Your Absolute Best Breathing Techniques Ever! on February 26th. They have indicated that they will be trying to make the past webinar available for viewing. [Information about how to register for future webinars then provided.]*

Finally, many reader-participants suffering from PF simply offered other PF patients supportive comments that appeared to be intended to validate their experience and shore up their resolve. For example, one blog reader-participant wrote in response to another’s comment:

*Loretta, I've looked into your story and am amazed at what you've been through. With your comorbitities, are you a candidate for a transplant?*

## Discussion

In this study, we analyzed posts on three PF-focused websites and found that PF patients and their loved ones engage the Web 2.0 environment to share their personal narratives about PF, to seek information from the PF community, and to offer contributions of various kinds to the PF community.

PF is a life-altering disease for both patients and their loved ones [1, 2]. Dyspnea forces patients to begrudgingly accept “new normals” of decreasing physical activity as the disease progresses; cough induces frustration, embarrassment and social isolation; supplemental oxygen imposes a number of constraints and adjustments in and out of the home; and the variability of—and inability to predict—disease behavior over time induces discomforting uncertainty in the face of an ever-present threat of death from PF progression.

Given the potentially devastating impact of the disease, it is no surprise that people affected directly or indirectly by PF yearn for support and information. For years, patients with various other conditions (and their caregivers) have gone online seeking connectedness (i.e., the chance to give and receive emotional support in the form of caring, sympathetic or encouraging messages) and the bi-directional exchange of information [9–11]—either general (e.g., about a disease process) or specific (e.g., advice about one’s personal circumstance) [12]. Our study confirms that PF patients and their loved ones do, too.

The ebb and flow of online support generally adheres to the social support activation model, which describes two dimensions of support-seeking behavior: 1) direct vs. indirect elicitation and 2) verbal vs. non-verbal elicitation [13]. People seeking emotional support online tend to tell narratives about their disease and their own emotional reactions to it, while people seeking informational support online tend to ask focused questions [14]. People provide emotional support online when they read personal disclosures about negative experiences or negative thoughts/feelings, and people tend to provide informational support online when they read questions, particularly those stated in the context of negative events [15]. Although it was not our intent to decipher whether posts from the PF community strictly adhered to these principles, they generally did. Some reader-participants used the sites to primarily receive support; others used them to give support; and many used them for both these (and still other) purposes.

Participation in online medical support communities decreases depression, increases both self-efficacy and quality of life [16] and empowers patients [17]. These benefits derive from meaningful conversations between patients struggling with similar problems. Web conversations also can boost morale with suggestions about how to achieve life goals within the confines of patients’ current health circumstances [18]. Besides providing a platform for patient-to-patient communication and support, the three PF-focused websites likely offer a sense of security and confidence in information to reader-participants, because they are hosted by clinicians who specialize in PF.

This study has certain limitations. We were able to glean only so much about the people who posted on the websites (e.g., by examining the gender of the pronouns they used), and there is no way to assess the fidelity of any information posted or the presence/extent of misinformation bias. Likewise, because we had no access to data on disease duration or severity, it is difficult to know whether our findings are applicable to all PF patients. This is a challenge faced by all researchers using these types of data. Of course, qualitative data can be gathered by other means, such as interviews. However, responses in interviews are built around memories and “narrative reconstruction of self,” and these can be greatly influenced by the interviewer and the setting [19]. Furthermore, communicating about illness involves more than rote question and answer; it includes exchanging information and emotional support [4]. Thus, online venues may provide qualitative data that are less biased and more authentic than those gathered using other methods.

There are many benefits to patients who engage the Web 2.0 environment. Participants can choose whether and when to respond to a post; they can raise issues and respond to posts that resonate with them in real time [4]. There are no transportation, geographic or disease severity barriers; patients with even the most severe PF can read, post, or both. Given the potential for anonymity, people can discuss sensitive issues online, such as physical appearance, intimacy, or public signs of illness (e.g., oxygen cannulas). However, because participants must necessarily have access to and familiarity with the internet [20], the reader-participants whose comments we analyzed may not be demographically representative of the PF population, particularly in terms of age, education, or affluence. Also, the three websites are written in English (and thus require English-language proficiency); this further limits the generalizability of our data to the universe of PF patients. However, unlike other information sources (e.g., pamphlets), the web offers immediate and bi-directional information exchange and a sense of community for those who share similar experiences. We encourage practitioners caring for PF patients to inform their patients of these sites and confidently recommend the sites to them as adjunctive sources of supportive and educational material.

## Conclusion

In conclusion, we analyzed posts on three PF-focused websites and found that patients and their loved ones engaged the Web 2.0 environment to seek and share information; i.e., to establish a reciprocal relationship with the PF community. Their posts and responses revealed their need to be both supportive and supported, their hunger for understanding PF from pathophysiology to prognosis, and their willingness to share experiences, knowledge and resources with the PF community.

## Additional file

Additional file 1:
**Online Supplement.** (DOCX 28 kb)

## Abbreviation

PF: pulmonary fibrosis

## References

[1] Swigris JJ, Stewart AL, Gould MK, Wilson SR (2005). Patients' perspectives on how idiopathic pulmonary fibrosis affects the quality of their lives. Health Qual Life Outcomes.

[2] Belkin A, Albright K, Swigris J (2013). A qualitative study of informal caregivers’ perspectives on the effects of idiopathic pulmonary fibrosis. BMJ Open Resp Res.

[3] Collard HR, Tino G, Noble PW, Shreve MA, Michaels M, Carlson B, Schwarz MI (2007). Patient experiences with pulmonary fibrosis. Respir Med.

[4] Seale C, Charteris-Black J, MacFarlane A, McPherson A (2010). Interviews and internet forums: a comparison of two sources of qualitative data. Qual Health Res.

[5] Robinson KM (2001). Unsolicited narratives from the Internet: a rich source of qualitative data. Qual Health Res.

[6] Graneheim UH, Lundman B (2004). Qualitative content analysis in nursing research: concepts, procedures and measures to achieve trustworthiness. Nurse Educ Today.

[7] Hsieh HF, Shannon SE (2005). Three approaches to qualitative content analysis. Qual Health Res.

[8] Charmaz K (2006). Constructing grounded theory: a practical guide through qualitative analysis.

[9] Braithwaite DO, Waldron VR, Finn J (1999). Communication of social support in computer-mediated groups for people with disabilities. Health Commun.

[10] Klemm P, Reppert K, Visich L (1998). A nontraditional cancer support group. The Internet. Comput Nurs.

[11] White MH, Dorman SM (2000). Online support for caregivers. Analysis of an Internet Alzheimer mailgroup. Comput Nurs.

[12] Ridings C, Gefen D. Virtual Community Attraction: Why People Hang Out Online. J Computer-Mediated Communication. 2004;10:00. doi:10.1111/j.1083-6101.2004.tb00229.x.

[13] Barbee A, Cunningham M, Winstead B, Derlega VJ, Gulley MR, Yankeelov PA, Druen PB (1993). Effects o gender role expectations on the social support process. J Soc Issues..

[14] Rodgers S, Chen Q (2005). Internet community group participation: psychosolial benefits for women with breast cancer. J Comput-Mediat Commun.

[15] Wang YC, Kraut RE, Levine JM (2015). Eliciting and receiving online support: using computer-aided content analysis to examine the dynamics of online social support. J Med Internet Res.

[16] Rains S, Young V (2009). A Meta-Analysis of Research on Formal Computer-Mediated Support Groups: Examining Group Characteristics and Health Outcomes. Hum Commun Res.

[17] Burrows R, Nettleton S, Pleace N, Loader B, Muncer S (2000). Virtual community care? Social policy and the emergence of computer mediated social support. Inf Commun. Soc..

[18] Wentzer H, Bygholm A (2010). Compliance or patient empowerment in online communities: reformation of health care services?. Stud Health Technol Inform.

[19] Williams G (1984). The genesis of chronic illness: narrative re-construction. Sociol Health Illn.

[20] White M, Dorman SM (2001). Receiving social support online: implications for health education. Health Educ Res.

