# Supplementary material for: Seeking and sharing: why the pulmonary fibrosis community engages the web 2.0 environment
Source: BMC Pulm Med. 2016 Jan 12;16:4. doi: 10.1186/s12890-016-0167-7 (PMC4709949; doi:10.1186/s12890-016-0167-7)
Supplement: Additional file 1: — Online Supplement. (DOCX 28 kb) [file 12890_2016_167_MOESM1_ESM.docx]

**ONLINE SUPPLEMENT**

**Seeking and sharing:**

**the pulmonary fibrosis community engaging the web 2.0 environment**

^1^Karen Albright, PhD

^2^Tarik Walker, MD

^3^Susan Baird, PhD

^3^Linda Eres

^3^Tara Farnsworth, MPH

^3^Kaitlin Fier, MPH

^3,4^Dolly Kervitsky

^3^Marjorie Korn

^5^David Lederer, MD

^3^Mark McCormick

^6^John F. Steiner, MD, MPH

^3^Thomas Vierzba

^3^Frederick S Wamboldt, MD

^3^Jeffrey J Swigris, DO, MS

^1^Department of Community and Behavioral Health, Colorado School of Public Health, University of Colorado School of Medicine; Aurora, Colorado

^2^Department of Pediatric Infectious Disease, University of Colorado School of Medicine; Aurora, Colorado

^3^Participation Program for Pulmonary Fibrosis;

National Jewish Health; Denver, Colorado

^4^PF Strategies, LLC; Black Hawk, Colorado

^5^Columbia University Medical Center; New York, New York

^6^Research Director, Kaiser Permanente Colorado

None of the authors has any conflict of interest (or competing interests) with the contents of this manuscript.

Author Correspondence:

Jeffrey J. Swigris, DO, MS

Associate Professor of Medicine

Director, Interstitial Lung Disease Program

National Jewish Health

1400 Jackson Street

Denver, Colorado 80206

Phone: (303) 398-1621

Fax: (303) 398-1040

email: swigrisj@njc.org

**METHODS
*Data Collection***

J.S.’s blog was started as part of the Participation Program for Pulmonary Fibrosis (P_3_F at

[www.PFresearch.org](http://www.PFresearch.org)), a nationwide research program funded by the Patient Centered Outcomes Research Institute (PCORI). D.L.’s blog ([www.PFdoc.org](http://www.PFdoc.org)) was started as a response to patient queries and general public interest in PF. The blogs and forum are accessible through a number of mechanisms, including internet searches, Facebook pages, Twitter accounts, an online support group supported by the Pulmonary Fibrosis Foundation (PFF), and notices posted on the websites for the PFF and the Coalition of Pulmonary Fibrosis. Readers of the blogs and forum are a self-selected group; participation is entirely voluntary; and reader-participants are free to maintain anonymity as they wish. On each blog, the pulmonary specialist or a member of his staff posts original blog entries and, where relevant, also posts replies to comments and questions posed by blog readers. On the forum, the topics of the original entries are generated completely by reader-participants; the facilitator (J.S. or D.L.), members of his staff, or other reader-participants may respond to those entries.

Data categories included in the Microsoft Excel database included the data source (i.e., which website), user name of poster, post date, post title, entire post content, number of responses to post, and number of unique user names, which serves as a proxy for number of unique reader-participants. For every response to a post and for each subsequent reply, data categories included the user name of responder, response date, status of responder (PF patient, caregiver, or facilitator), and entire content of response. All data are online and of public record; therefore, Institutional Review Board (IRB) approval for this study was not required.
